# Stem cells as a therapeutic tool for the blind: biology and future prospects

**DOI:** 10.1098/rspb.2011.1028

**Published:** 2011-08-03

**Authors:** Mandeep S. Singh, Robert E. MacLaren

**Affiliations:** 1 Nuffield Laboratory of Ophthalmology, University of Oxford, Oxford OX3 9DU, UK; 2 Merton College, University of Oxford, Oxford OX1 4JD, UK; 3 Moorfields Eye Hospital, London EC1V 2PD, UK; 4 Oxford Eye Hospital, Oxford OX3 9DU, UK

**Keywords:** age-related macular degeneration, retinitis pigmentosa, retinal degeneration, embryonic stem cell, induced pluripotent stem cell, transplantation

## Abstract

Retinal degeneration due to genetic, diabetic and age-related disease is the most common cause of blindness in the developed world. Blindness occurs through the loss of the light-sensing photoreceptors; to restore vision, it would be necessary to introduce alternative photosensitive components into the eye. The recent development of an electronic prosthesis placed beneath the severely diseased retina has shown that subretinal stimulation may restore some visual function in blind patients. This proves that residual retinal circuits can be reawakened after photoreceptor loss and defines a goal for stem-cell-based therapy to replace photoreceptors. Advances in reprogramming adult cells have shown how it may be possible to generate autologous stem cells for transplantation without the need for an embryo donor. The recent success in culturing a whole optic cup *in vitro* has shown how large numbers of photoreceptors might be generated from embryonic stem cells. Taken together, these threads of discovery provide the basis for optimism for the development of a stem-cell-based strategy for the treatment of retinal blindness.

## 1. Introduction

A recent publication in this journal described how an electronic device implanted under the human retina could restore vision to a blind eye [1]. One patient was able to read large print despite years of visual impairment prior to implantation. This finding and others confirm that a retina that has lost all light-sensitive cells—the photoreceptors—might regain function if residual neurons are stimulated by new light-sensing components, despite neuronal and glial reorganization [2–4]. The discovery that a device implanted under the retina can achieve vision restoration is significant because the stimulus is placed where the original light-sensitive cells would have been located, taking advantage of downstream processing that occurs in other retinal neurons before signals reach the visual cortex. This finding supports the development of stem cell treatments to replace photoreceptors as such treatments would be delivered to this same anatomical location.

**Figure 1.**
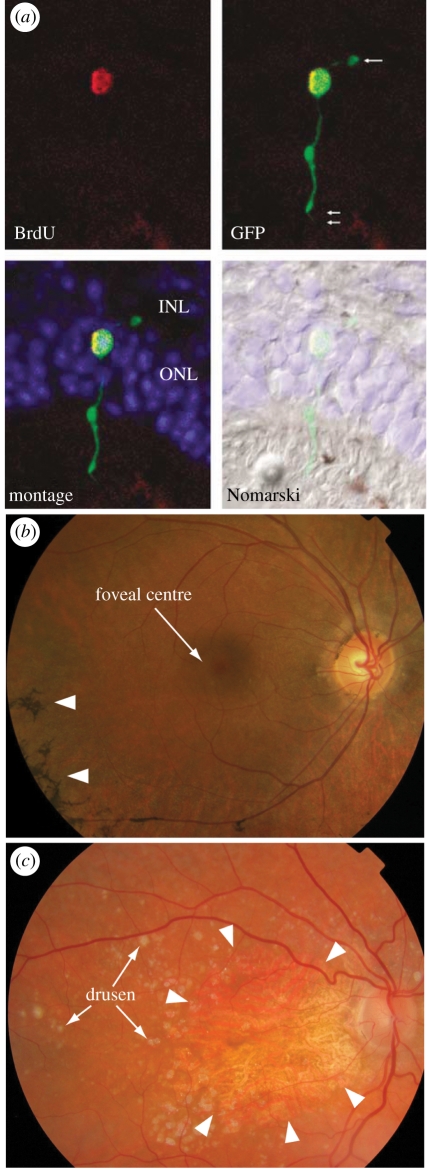
Outer retinal degenerations may in future be amenable to cell replacement. (*a*) Successful integration of a donor photoreceptor, expressing green fluorescent protein (GFP; green) and labelled with bromodeoxyuridine (BrdU), into a host outer nuclear layer (ONL). INL, inner nuclear layer (reproduced with kind permission from MacLaren & Pearson [6], © 2007 Nature Publishing). (*b*) Retinitis pigmentosa (here, Thr17Met rhodopsin mutation) starts in the retinal periphery, with pigmentary changes (arrowheads) owing to degeneration of the outer retina. The disease commonly proceeds to threaten the foveal centre and will then affect central vision. (*c*) Dry age-related macular degeneration is characterized by white deposits called drusen. Here a large, central patch of retinal pigment epithelium and outer retinal atrophy is seen (margins demarcated with arrowheads), and would be consistent with markedly reduced visual acuity.

In cell replacement therapy for most central nervous system (CNS) diseases, replacement neurons would need to develop afferent and efferent connections with the host. In some cases, the replacement neurons would need to navigate across long distances, and it is unlikely that the necessary axon guidance cues would persist in the adult brain [5]. Blindness in retinal degenerations is caused by death of the first neuron in its pathway: the photoreceptor. Photoreceptors, stimulated by photons, are not dependent on afferent synapses. Replacement cells (figure 1*a*) would need to make only one efferent connection with an adjacent second-order neuron in the host inner retina with no need for navigation, to re-establish the visual circuit. It is therefore arguable that the photoreceptor is among the most readily transplantable neurons in the CNS, and is an excellent candidate for clinical trials exploring regenerative neural stem cell therapies.

## 2. The clinical need

Age-related, diabetic and genetic retinal degenerations account for over 50 per cent of blind patients in the developed world [7]. Retinitis pigmentosa (RP) is the leading cause of inherited retinal blindness in younger patients, with a prevalence of approximately 1 : 4000 [8]. Retinitis pigmentosa is a term encompassing a group of disorders, caused by mutations in over 150 genes discovered to date [9], that cause photoreceptor loss which typically progresses to involve the central retina at which point all sight is lost (figure 1*b*).

Age-related macular degeneration (AMD) is a common disease with genetic and environmental risk factors [10,11]. AMD affects 14 million elderly people in the developed world [11,12]. Ninety per cent of patients suffer from the form termed dry AMD, which is characterized by primary degeneration of retinal pigment epithelium (RPE) cells leading to secondary photoreceptor loss [13] and is currently untreatable (figure 1*c*).

One other retinopathy has attracted attention for stem cell therapy. Stargardt disease is the most common inherited juvenile macular degeneration [14]. Symptoms begin typically between the ages of 6 and 12, with a variably progressive course. In November 2010, Advanced Cell Technology (ACT), a US-based company, announced that they had gained regulatory approval to use embryonic stem (ES) cells to replace RPE in a phase I/II clinical trial involving patients with Stargardt disease (http://www.advancedcell.com/news-and-media/press-releases/advanced-cell-technology-receives-fda-clearance-for-the-first-clinical-trial-using-embryonic-stem-cel/, accessed 5 April 2011). In this proposed therapy, treatment will target RPE replacement to restore function and/or prevent loss of photoreceptors. However, once photoreceptors are lost in advanced degeneration, treatment must involve reintroduction of light-sensitive cells. The challenge for retinal stem cell therapy lies in the generation of numerous photoreceptor cells, at the ideal developmental stage to integrate, free of malignant potential and immunogenicity. Although close, this has not yet been achieved to a level sufficient to consider clinical trial approval; the on-going work with RPE will provide valuable safety data to support future trials using stem cell approaches to replace photoreceptors.

## 3. The retina: an ideal testing ground for neural regeneration

**Figure 2.**
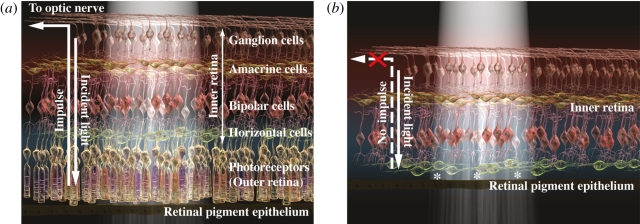
(*a*) Normal retina. Photoreceptor cells are the light sensors in the visual system. In the laminations of the normal human retina shown here, photoreceptors lie in the outer retina, optimally oriented to detect the incidence of photons. The photoreceptors have a light-sensitive outer end that is apposed to retinal pigment epithelium (RPE), and an inner end specialized for synaptic transmission that is connected to second-order neurons in the inner retina. When stimulated, photoreceptors generate impulses that are processed by inner retinal cells and then conveyed to the brain via the optic nerve. Visual sensation is produced when impulses reach the visual cortex in the occipital lobe. (*b*) Severe retinal degeneration. In the diagram above, all photoreceptors have been lost and the retina is unable to sense incident light. There are no signals conveyed to the inner retina and brain, and the patient is therefore blind. Retinal sensitivity may, in principle, be restored by placing new light-sensing components, such as stem/precursor cells or an electronic prosthetic device, between host inner retina and host RPE (location marked by asterisks), where photoreceptors would normally be located. In this way, inner retinal processing is used to optimize the signals generated from the new light sensors before these are transmitted to the cortex. (Reproduced with kind permission from MacLaren & Pearson [6], © Nature Publishing.)

The human retina contains rod photoreceptors for vision in low light and cone photoreceptors for colour and high-acuity vision in bright light. Photoreceptors depend on RPE for metabolic activity (figure 2). Also, cones are dependent for survival on the rods through a number of putative mechanisms, including paracrine support [15]; the loss of one class of cell therefore leads to the secondary loss of others. Hence, cell replacement strategies in RP might aim to reintroduce rods in order to rescue cones from degeneration, or alternatively to restore night vision directly.

Retinal cell therapy is likely to be delivered using small-gauge vitrectomy, a procedure now routinely performed on an outpatient basis with low morbidity. The range of assessments for retinal structure and function can provide the safety and efficacy data required in clinical trials. Confocal scanning laser ophthalmoscopy reveals retinal structure to a resolution of 7 µm [16] and may be employed to visualize cell grafts. Whereas grafts in other organs may be difficult to visualize, they may easily be monitored in the retina through the clear cornea and lens. As uncontrolled proliferation is a concern for any stem cell treatment, graft site visualization is a distinct advantage in terms of safety. Also, cells would be transplanted into the subretinal space, a discrete compartment that would limit the systemic spread of immature cells. Laser photocoagulation—used routinely in advanced diabetic retinopathy—could be used if necessary to destroy transplanted cells non-invasively. A range of tests including visual electrophysiology, microperimetry, contrast sensitivity and mobility testing (among others) are available to assess visual function and treatment efficacy even when only a minimal degree of function remains, as will be the case in patients requiring therapy. Furthermore, the fellow eye provides a control as retinal degenerations are frequently bilateral and symmetrical.

## 4. Challenges in retinal replacement

The first report of mammalian retinal transplantation by Katharine Tansley [17] initiated decades of interest in the use of immature tissue for retinal replacement [18–22]. Photoreceptor-related functions are known to occur in embryonic and foetal retinal cells upon maturation [23,24], and provide the basis to be hopeful for the development of functional photoreceptors from immature cells.

**Figure 3.**
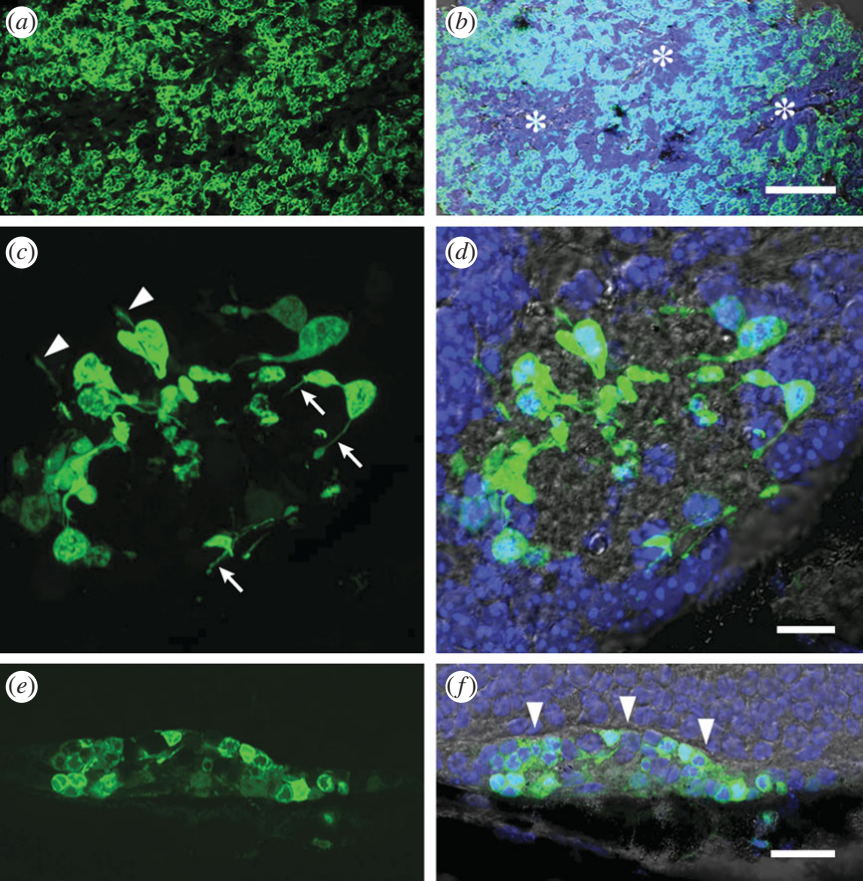
Challenges in cell-based retinal therapy. (*a*,*b*) Rosette formation hampers integration of transplanted precursor cells. Here, retinal cells from *nrl*.*gfp*^+/+^ mice (see [33]) that express green fluorescent protein (GFP; green) in post-mitotic rod precursors were dissociated before subretinal transplantation. Rosettes (asterisks) formed after transplantation, and there was poor donor–host integration. Scale bar, 50 µm. (*c*,*d*) Accurate labelling of host cells is important to interpret morphology. GFP-positive donor cells extend processes from the cell bodies. Whereas some processes (arrows) appear to extend within the graft, others (arrowheads) appear to point towards host tissue. However, without a histological marker for host cells, synaptic connections of donor and host are difficult to demonstrate. Scale bar, 10 µm. (*e*,*f*) Integration may be prevented by gliosis in degenerated hosts. Here, GFP-positive cells do not extend processes into the host retina, and a sharp demarcation is seen (arrowheads) between the donor cell mass and host retina, presumably owing to advanced gliosis. Scale bar, 25 µm. (*b*,*d*,*f*) The merged GFP and transmitted light channels. Nuclei are counterstained with Hoechst-33342 (blue).

For successful integration, the grafted photoreceptors should assume the correct orientation, with an inner synapse and an outer photoreceptive segment positioned against host inner retina and RPE, respectively. To this end, different methods of donor cell preparation have been proposed. Whole retinal sheets derived from embryonic or neonatal rodents can survive and differentiate after subretinal transplantation [25–27]. A recent study employed attenuated pseudorabies virus to label graft neurons and showed that full-thickness retinal sheets, while not integrating directly, could connect with host neurons [28]. Similar observations have been made with partial-thickness sheets [29,30]. A lack of integration is likely to be more of a problem when using a single-cell suspension as orientation will be significantly disrupted, and, furthermore, there is a tendency for rosette formation [31,32]. These cells would be unable to generate visual responses if aggregations directed light-induced currents away from host retina (figure 3*a,b*). The relative ease of surgical delivery, however, has made the use of single-cell suspensions appealing and it has been widely investigated [32,34,35], with evidence that some cells integrate into host retina and make synaptic connections [36]. Currently, the percentage of cells able to integrate and make connections is low (approximately 0.1 per cent) [37]. Given that millions of photoreceptors will probably be needed to restore meaningful vision, the challenge for the cell suspension approach will be to generate sufficient numbers of cells and in parallel to improve integration efficiency.

Positioned in the subretinal space, the immature cell must develop an inner process specialized for synaptic transmission to second-order neurons. Evidence of neural integration following transplantation has been presented [30,38–43]; however, as typified by these studies, accurate discrimination of donor and host is a challenge because graft–host synapses are difficult to distinguish from intra-graft synapses (figure 3*c,d*). Notably, synapses may not be necessary as residual retinal cells may, in theory, be stimulated by a proximate potential change, as exemplified by the retinal implant device.

Transplantation into the retina may be hampered by gliosis (figure 3*e,f*), although this may be less marked than other CNS sites [44–46]. Disruption of glial barriers may result in better integration [37,47,48]. Clinically, it is recognized that intra-retinal RPE migration is a feature of RP, which may imply that gliosis could still allow for cell integration to some extent.

A critical translational question is whether transplanted stem or precursor cells will improve function in a degenerate host. Hosts that still have at least some degree of outer retinal architecture have shown functional improvement following photoreceptor replacement [36,49–53]. However, it is not known whether vision can be restored in a severely degenerate retina with prolonged photoreceptor loss. It will be critical to address this in order to apply these treatments in patients with severe blindness.

Clinical trials have shown that the human foetal retina can be transplanted into the subretinal space without significant surgical adverse reactions [54] or immune rejection [55]. However, another report presented angiographic evidence of inflammation after a similar transplant [56], with no effect on vision. Nevertheless, these pioneering studies show that transplantation of immature tissue into the severely degenerated retina in humans is a safe procedure, so the question of whether such transplants can restore sight may soon be answered.

## 5. Sources of cells for transplantation

ES cells, derived from the inner cell mass of blastocyst-stage embryos [57–59], are able to maintain an undifferentiated state or can be directed to mature along lineages deriving from all three germ layers—ectoderm, endoderm and mesoderm. Photoreceptor features were found in subretinal human ES cell grafts, but not in locations elsewhere in the eye, indicating that the subretinal niche may be critical to support differentiation of ES cells towards a photoreceptor fate [60]. Retinal fate has also been induced in mouse, monkey and human ES cells by using growth factors, retinal co-culture and genetic modification [61,62]. When directed to become retinal precursors similar to the human foetal stage, human ES cells were found to integrate into an explant model of Leber congenital amaurosis [63] and restore some function *in vivo* [64]. These data strongly support the use of precursor cells for photoreceptor replacement. It may be that for effective integration, stem cells need to be differentiated some way along the photoreceptor lineage before transplantation; recent evidence has suggested that even mature neurons may integrate [65]. Very recently, it was shown that three-dimensional culture of mouse ES cell aggregates led to autonomous optic cup formation *in vitro* with features of retinal stratification [66]. A similar system might be applicable to expand and differentiate a single ES cell into potentially thousands of photoreceptor precursors (or some other photoreceptor developmental stage) for optimal integration.

Despite the relative ocular immune privilege, ES cells are immunogenic as they originate from another human foetus, which also raises ethical questions about how to source these cells. The discovery that reprogramming DNA-binding proteins may induce stem cells from adult cells represents a milestone in the search for a renewable source of cells [67–69]. These ‘induced pluripotent’ stem (iPS) cells, being autologous, may obviate the need for chronic immune suppression.

iPS clones could be derived from patients and be used for treatment—a process that may involve *ex vivo* correction of the gene defect before reintroduction into the host. Recent progress has been made as iPS cells derived from amyotrophic lateral sclerosis patients have been differentiated into motor neurons—the cell type that requires replacement in this condition [70]. In a similar vein, human iPS cells have been cloned from Parkinson's disease patients [71] and iPS cell-derived dopamine neurons have improved function in a Parkinson's disease model [72]. However, iPS clones vary in pluripotency and differentiate less efficiently than ES cells, which show robust neuronal differentiation [73]. Interestingly, this variability is independent of the type of vector used in iPS cell production. Integrating vectors such as lentiviruses (whereby genes are inserted into the target cell genome), in addition to affecting pluripotency, confer a greater potential risk of teratogenicity than non-integrating vectors (whereby the gene is expressed while remaining separate from the host genome). By avoiding the use of genes and vectors associated with uncontrolled proliferation [74,75], the risk of tumour formation is reduced. Ideally, iPS cells derived from every patient will need to be screened for potentially cancerous cells, as even a 0.01 per cent risk of malignancy induced by therapy may be unacceptable for patients and doctors. The situation is somewhat different with ES cells, which are not derived from individual patients—potentially, a few well-characterized, purified and approved cell lines may be used widely. Overall, the regulatory environment will be complex, given the range of pluripotency, differentiation capacity, teratogenicity and immunogenicity of different iPS cell clones and ES cell lines. It is likely that a range of pre-treatment protocols, potentially subject to differing regulatory requirements, will be developed in future, tailored to specific clinical situations.

**Figure 4.**
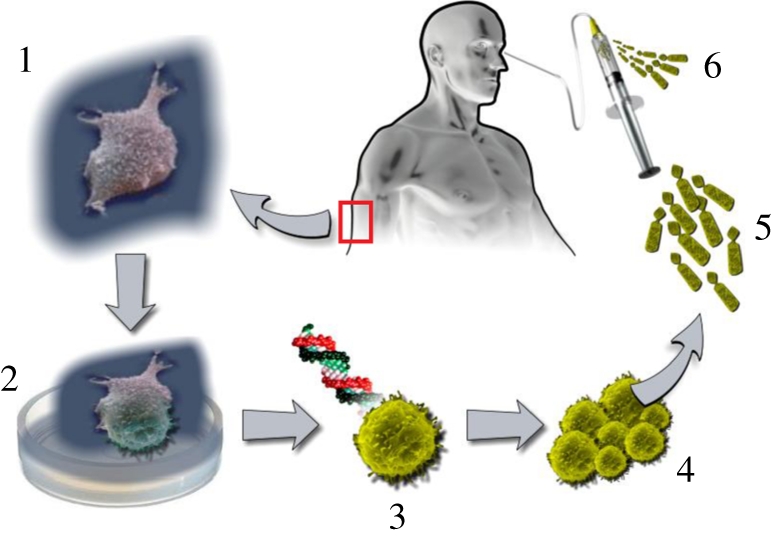
1. A suitable cell is obtained from the patient with retinal disease. An easily accessible source of cells is chosen so as to minimize surgical trauma. The example of a skin fibroblast is depicted here. 2. The capacity for self-renewal—absent in almost all adult cells—is restored *in vitro* through genetic reprogramming or other means, so that this cell may be expanded in numbers later on. In theory, only one reprogrammed cell is required to repopulate any diseased tissue. Reprogramming methods should be chosen that minimize the risk of mutagenesis and uncontrolled proliferation. 3. The genetic mutation that caused blindness in this patient could, in principle, be corrected at this stage by *ex vivo* gene therapy. Other modifications may also be added to enhance cell survival and integration in the host. 4. This genetically corrected autologous cell is expanded *in vitro* to produce the large number of cells required for a therapeutic effect. Preferably, products derived from animals or bacteria are not used in the cell culture protocol. 5. The cells are made to develop up to a stage known to lead to optimal integration and function upon transplantation. The resultant colony must ideally be free of malignant potential and immunogenicity. 6. The cells are delivered to the subretinal space using well-established surgical techniques with low morbidity, such as small-gauge vitrectomy.

iPS cells have differentiated *in vitro* into retinal cell phenotypes, recapitulating events in normal development [76,77]. Functional human photoreceptor-like cells have been observed following differentiation of human iPS cells [78,79]. In the case of retinal degenerations, iPS cells from patients might be used for replacement, with correction of the RP gene defect and the cells then directed to assume a retinal precursor fate before transplantation (figure 4). For therapy, abundant autologous cells should be generated at a stage with maximal integrative capacity. For regulatory purposes, it would be optimal to use protocols that do not rely upon materials of bacterial or animal origin; low-molecular-weight compounds have been shown to induce retinal progenitors, RPE and photoreceptors from ES and iPS cells [80].

## 6. Retinal pigment epithelium cell transplantation

In AMD, the primary cell to be lost is the RPE cell, leading to a secondary loss of photoreceptors. Hence, it has been asked if RPE replacement may delay or prevent blindness in AMD. Photoreceptor rescue has been reported after transplantation of foetal RPE cells in animals, even in advanced disease [81]. This approach has not yet, however, shown convincing effect in AMD patients [82,83]. This is in contrast to autologous grafts, which included Bruch's membrane, an underlying platform that maintains RPE polarity and homeostasis [84,85]. Hence, the challenge in using stem cells for RPE replacement will probably be to find a means of integrating differentiated RPE cells with a basement membrane—recreating cell adhesion junctions to Bruch's membrane or an alternative substrate, rather than creating synapses with host neurons.

In searching for renewable RPE cells, RPE-like cells have been derived from non-human primate and human ES cells [86–88]. Human ES-cell-derived RPE has been shown to improve retinal function in a rat model of AMD [89–91], and iPS-cell-derived RPE was found to have protective effects in dystrophic rats [92]. Human ES-cell-derived RPE cells have been studied for prolonged periods and found to sustain function without evidence of teratoma formation [93], paving the way to clinical application. The potential commencement of the recently announced phase I/II trial using ES cells for RPE replacement, discussed earlier, will encourage the development of the ideal cell for translation into AMD patients.

## 7. Conclusion

The clinical need for therapies for retinal degeneration has energized the search for renewable cells to replace photoreceptors in patients. It is known that the diseased human retina may recover function if appropriately stimulated by an electrical current, and separately it has been demonstrated that rod photoreceptor precursor cells may mature, differentiate and function in the host environment after transplantation. Moreover, large numbers of cells may potentially be generated from a single stem cell in an optic cup culture model. The convergence of these discoveries sets the foundation in the near future for a clinically applicable stem cell strategy to restore vision in patients with retinal degenerations.
